# Reusability of SPE and Sb-modified SPE Sensors for Trace Pb(II) Determination

**DOI:** 10.3390/s18113976

**Published:** 2018-11-15

**Authors:** Matjaž Finšgar, David Majer, Uroš Maver, Tina Maver

**Affiliations:** 1 Faculty of Chemistry and Chemical Engineering, University of Maribor, Smetanova ulica 17, 2000 Maribor, Slovenia; david.majer@student.um.si; 2 Faculty of Medicine, Institute of Biomedical Sciences, University of Maribor, Taborska ulica 8, SI-2000 Maribor, Slovenia; uros.maver@um.si; 3 Faculty of Mechanical Engineering, University of Maribor, Smetanova ulica 17, 2000 Maribor, Slovenia; tina.maver@um.si

**Keywords:** screen-printed electrode, Sb-film electrode, SPE, SbFE, stripping analysis, EIS

## Abstract

In this work, unmodified screen-printed electrode (bare SPE) and Sb-film modified SPE (SbFSPE) sensors were employed for the analysis of trace amounts of Pb(II) in non-deaerated water solutions. The modified electrode was performed in situ in 0.5 mg/L Sb(III) and 0.01 M HCl. The methodology was validated for an accumulation potential of –1.1 V vs. Ag/AgCl and an accumulation time of 60 s. A comparative analysis of bare SPE and SbFSPE showed that the detection and quantification limits decrease for the bare SPE. The method with the bare SPE showed a linear response in the 69.8–368.4 µg/L concentration range, whereas linearity for the SbFSPE was in the 24.0–319.1 µg/L concentration range. This work also reports the reason why the multiple standard addition method instead of a linear calibration curve for Pb(II) analysis should be employed. Furthermore, the analytical method employing SbFSPE was found to be more accurate and precise compared to the use of bare SPE when sensors were employed for the first time, however this performance changed significantly when these sensors were reused in the same manner. Furthermore, electrochemical impedance spectroscopy was used for the first time to analyse the electrochemical response of sensors after being used for multiple successive analyses. Surface characterisation before and after multiple successive uses of bare SPE and SbFSPE sensors, with atomic force microscopy and field emission scanning electron microscopy, showed sensor degradation. The interference effect of Cd(II), Zn(II), As(III), Fe(II), Na(I), K(I), Ca(II), Mg(II), NO_3_^–^, Bi(III), Cu(II), Sn(II), and Hg(II) on the Pb(II) stripping signal was also studied. Finally, the application of SbFSPE was tested on a real water sample (from a local river), which showed high precision (RSD = 8.1%, *n* = 5) and accurate results.

## 1. Introduction

The natural environment and the environment polluted by humans contain different types of heavy metals. Monitoring of trace heavy metals is extremely important in order to prevent their bioaccumulation and other health-related problems. Such analyses are usually carried out using spectroscopic techniques, such as inductively coupled plasma mass spectrometry (ICP-MS), inductively coupled plasma optical emission spectrometry (ICP-OES), and atomic absorption spectrometry (AAS). An alternative is electroanalytical analysis, which is advantageous, not only due to its low instrumentation and analysis costs, but also because it enables on-site analysis, directly after the sampling is carried out (e.g., from a lake, river, etc.). Among the electroanalytical techniques, anodic stripping voltammetry (ASV) is among the most effective due to a pre-concentration step enabling trace or ultra-trace analysis of different heavy metals with high sensitivity, accuracy, and precision [1]. 

This work is focused on the detection of Pb(II), a heavy metal with proven toxicity to humans. The usual consequences of Pb uptake can be observed in gastrointestinal, neuromuscular, and neuropathological symptoms. These are most severe after longer exposure times and are especially serious in children below six years of age, whose mental and physical development can consequently decrease. Pb poisoning can also be fatal [2]. Considering everything mentioned above, it is clear that a disposable, low cost, and portable sensor for Pb(II) detection could be highly beneficial for human health. 

Modified electrodes, especially glassy carbon electrodes (GCE), have recently been replacing classic polarography, which uses Hg-based electrodes. In such a manner, mercury waste, following analysis, is avoided—in accordance with the Minamata Convention on Mercury, which has been signed by 128 countries in order to protect human health [3]. Another advantage of some modified electrodes is that they eliminate the need for oxygen removal, which is a necessary and time-consuming step in polarography. Various modified electrodes have been reported, whereas the bismuth-film glassy carbon electrode (BiFE) seems to be one of the best substitutes for the Hg electrode in the electroanalytical determination of traces of heavy metals [4,5,6]. On the other hand, other substitutes are currently being sought [7]. Among them, antimony-film GCE (SbFE) is another attractive substitute for polarography and even for BiFE [8,9,10,11,12,13,14,15,16]). Antimony is significantly less toxic than mercury and therefore much more acceptable for the environment [17,18]. For example, according to the German Water Hazard Class (WGK), the Sb(III) standard solution employed herein corresponds to the WGK1 that classifies this chemical as being relatively harmless and close to non-hazardous. Despite the relatively low number of studies in the last decade, this research area is still very attractive due to the advantages compared to classic polarography-based methods and as such is currently being extensively explored. For example, the advantage of using SbFE over BiFE lies in the analysis of Cu(II) traces [19], its wider potential window, the low stripping signal for Sb, the low background contribution, and the possibility of working in more acidic solution with pH ≤ 2 [8,10,12]. The above-mentioned electrodes are usually employed in combination with the ASV technique. 

Another field in electroanalytical research that is currently expanding is the use of screen-printed electrodes (SPE). The advantages of using SPEs over a conventional three-electrode configuration (working, reference, and counter electrodes) are the more convenient setup and the miniaturisation of the system, which facilitates portability. SPE electrodes are widely available from different producers. These electrodes can also be manufactured in an ordinary research laboratory on site by using inexpensive screen-printing equipment, a platform (e.g., ceramic of plastic), and a special ink. Compared to GCE, no polishing step is carried out before the analysis with the SPE [20,21], which makes these electrodes far more convenient. By combining the above-mentioned technologies, a new system can be developed, i.e., the antimony-film screen-printed electrode (SbFSPE). Currently, the most used working electrode platform material for SPE is carbon [8]. Sb-film can be electrodeposited on the working electrode in situ during the pre-concentration step or ex situ before the pre-concentration step [8,12,13,19,22,23,24]. SPE technology using different working electrodes for Pb(II) determination was employed previously [8]. Table 1 shows how the working electrode material affects the performance for some differently modified SPEs compared with the present work. Furthermore, not only inorganic modified electrodes perform well for heavy metal determination, but also some organic-based modified electrodes were developed for this purpose [25,26].

Despite being disposable, many SPE manufacturers claim that these sensors can be employed many times before being discarded. Moreover, Perez-Rafols et al. [13] and Sosa et al. [12] concluded that applications of these types of electrodes can be used for a large set of measurements without sign of degradation or loss of sensitivity. Hitherto, to the best of our knowledge, none of the available reports performed a systematic study of disposable SbFSPE sensor electrode degradation and performance after reuse. Evaluation of the possible reusability of such electrodes is one of the main focuses of the present study. Moreover, the evaluation of SbFSPE sensors has never before been evaluated using electrochemical impedance spectroscopy (EIS). In this work, square-wave anodic stripping voltammetry (SWASV) was used to analyse Pb(II) using bare SPE or SbFSPE. These sensors were also microscopically investigated before and after the analytical work. Finally, the interference effect of various ions was checked and the applicability of SbFSPE to real water samples was checked.

## 2. Materials and Methods

All of the electrochemical experiments were performed with a PGSTAT204 potentiostat/ galvanostat controlled by Nova 2.1.3 software (Metrohm Autolab B.V., Utrecht, The Netherlands) under laboratory conditions (23 °C ± 2 °C). Where applicable, outliers were checked with both Dixon’s and Grubbs’ statistical tests with 95% confidence [29], but none were detected, and, where applicable, the average values are reported.

### 2.1. Electrodes

For partial method optimisation and the determination of the optimum accumulation potential (*E*_accu_), a glassy carbon electrode (GCE) with a diameter of 3 mm (Cat. No. 6.1204.300) and a Pt wire counter electrode was used. All the potentials in this work refer to an Ag/AgCl electrode filled with saturated KCl (the potential of this reference electrode is 0.197 V vs. standard hydrogen electrode, SHE, at 25 °C). GCE, Ag/AgCl, and Pt electrodes were supplied by Metrohm (Herisau, Switzerland). Polishing of the GCE was performed with 0.05 µm Al_2_O_3_ (Buehler, IL, USA), followed by washing in ultra-pure water, cleaning for 5 min in an ultrasound bath containing ultra-pure water, and drying (with a paper towel without touching the active surface).

SPE sensors (DRP-110) were supplied by DropSens (Llanera, Spain). These sensors were constructed of carbon-based working (4 mm in diameter) and counter electrodes, and an Ag pseudo reference electrode. In all experiments using SPE sensors an external Ag/AgCl electrode was employed instead of an Ag pseudo reference electrode to better control the reference potential. The reversibility of the SPE sensor was checked with a cyclic voltammetry (CV) test for the potassium hexacyanoferrate system (results are given in the Supporting Information—Figure S1). 

### 2.2. Solutions and Reagents

All of the analytical work was performed in a 0.01 M HCl supporting electrolyte. HCl and KCl were supplied by Carlo Erba Reagents (Val de Reuil, France). Atomic absorption standard stock solutions (1000 mg L^–1^) of Pb(II), Sb(III), Cd(II), Zn(II), Sn(II), Bi(III), Cu(II), and As(III) were purchased from Merck (Darmstadt, Germany). (NH_4_)_2_Fe(SO_4_)_2_·6H_2_O, NaCl, and KNO_3_ were purchased from Sigma Aldrich (St. Louis, MO, USA), while CaCl_2_ and MgCl_2_ were supplied by Acros Organics (Morris, NJ, USA). All chemicals were of analytical grade purity. Ultra-pure water with a resistivity of 18.2 MΩ cm was employed to prepare all solutions (prepared using the Milli-Q water purification system, Millipore Corporation, Burlington, MA, USA). 

#### Real Sample Analysis

A river water sample was collected in the middle of a riverbed of the Drava River, which passes through Maribor (Slovenia) at the Lent site. A mixture of river and ultrapure water in a 1:5 ratio was prepared. This mixture was then used to prepare a 0.01 M HCl solution containing 0.5 mg/L Sb(III). The concentration of the unknown Pb(II) was determined using the multiple standard addition method (6 additions). By using Whattman glass fibre filters (0.45 µm pore size), the water sample was filtered after preparation and before analysis [13].

### 2.3. SWASV Experiment

The *E*_accu_ was set at −1.1 V for 60 s (pre-concentration step), followed by 15 s equilibration time, before starting the SWASV experiment. The SWASV measurements were performed using a positive-going square-wave potential scan in the potential region from −1.1 V to 0.3 V, with a potential step of 4 mV, an amplitude of 25 mV, and a frequency of 25 Hz [4]. After each SWASV experiment, a cleaning cycle was performed by applying a potential of 0.3 V for a period of 30 s. During the accumulation and cleaning steps, the solution was stirred, whereas during the equilibration and measurement steps no stirring was employed. All the CV and SWASV experiments were performed without deaeration of the solutions.

### 2.4. Electrochemical Impedance Spectroscopy 

EIS measurements were performed in the 10^6^–5 × 10^–2^ Hz frequency range using an excitation signal with an amplitude (peak-to-peak) of 10 mV. Five points were measured in each frequency decade. A pre-concentration step (the same as for the Pb(II) analysis, as described in Section 2.3) was performed before EIS analysis was carried out. EIS measurements were performed at −1.1 V. This procedure was performed for both bare SPE and SbFSPE sensors.

### 2.5. ICP-OES 

An ICP-OES instrument, i.e., Agilent 5110 VDV (Santa Clara, CA, USA), was used for the determination of Pb(II) in the real water sample. The gas employed was argon with 99.9999% purity (Messer, Maribor, Slovenia). The calibration curves were performed in the presence of yttrium as an internal standard.

### 2.6. Surface Analysis

The surface morphology of the working electrode surfaces were analysed by field emission scanning electron microscopy (FE-SEM). Prior to imaging, the sensors were pressed on double-sided adhesive carbon tapes (SPI 116 Supplies, West Chester, PA, USA). Micrographs were taken using FE-SEM (Supra 35 VP, Carl Zeiss, Oberkochen, Germany) operated at 1 keV at room temperature [30].

The surface topography and roughness parameters of the sensors were measured using an atomic force microscope (AFM) in tapping mode (7500 AFM model, Keysight, Santa Barbara, CA, USA). Silicon cantilevers (ATEC-NC-20, Nanosensors, Neuchâtel, Switzerland) with a resonance frequency of 210–490 kHz and a force constant of 12–110 N m^–1^ were used for all measurements, which were performed at room temperature. All surfaces were scanned with spot sizes of 10 × 10 μm^2^, recorded with a resolution of at least 512 × 512 lines [31]. PicoImage software (Keysight) was used to process all images and to calculate the corresponding roughness parameters [32]. 

## 3. Results and Discussion

Hereinafter, the term SbFSPE shall be used for the Sb-film SPE electrode formed in situ using an SPE sensor immersed in 0.01 M HCl containing 0.5 mg/L Sb(III). Thus, in situ Sb-film along with Pb is accumulated on the working electrode during the accumulation step (pre-concentration). The term bare SPE shall hereinafter stand for the system, where the Pb(II) analysis was performed in 0.01 M HCl without Sb(III) in the solution. The 0.01 M HCl supporting electrolyte was chosen in accordance with the first report on the use of an in situ Sb-film formed GCE electrode (SbFE) in that medium [10]. Furthermore, it has been reported that HCl stabilises antimony film [19]. For both SbFSPE and bare SPE, all Pb(II) measurements were performed at *E*_accu_ = −1.1 V and *t*_accu_ = 60 s (as shown below this accumulation time provided relatively wide linear concentration ranges, which would became narrower in case of using longer accumulation times [33]).

### 3.1. Method Validation 

**Figure 1:**
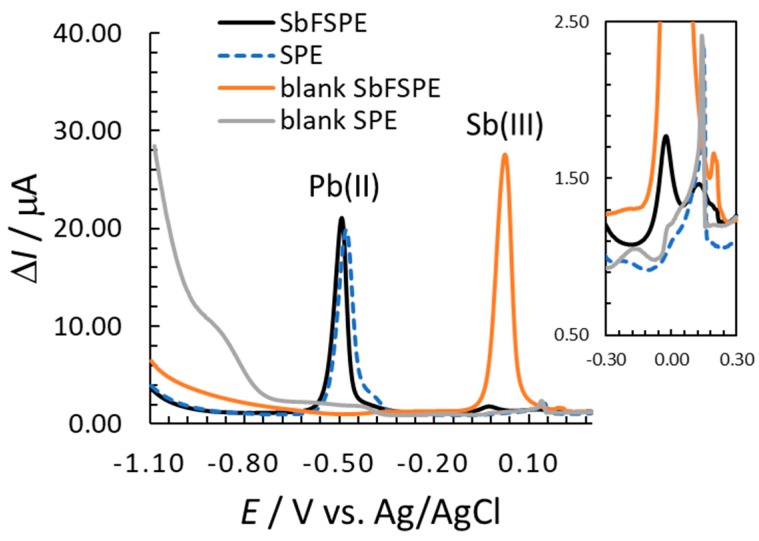
Blank measurements, stripping signals of Pb(II) and Sb(III) using SbFSPE, and stripping signals of Pb(II) for bare SPE in 0.01 M HCl; *γ*_Pb(II)_ = 164.8 µg/L (for SbFSPE and SPE measurements), *γ*_Sb(III)_ = 0.5 mg/L (in the case of SbFSPE), *E*_acc_ = −1.1 V, and *t*_acc_ = 60 s were employed.

Figure 1 shows SW voltammograms measured in 0.01 M HCl by employing SbFSPE and bare SPE sensors. The terms *γ* and ∆*I* stand for the mass concentration and the square-wave current signal, respectively. Hydrogen evolution starts at potentials more negative than −1.1 V (whereas it is not yet intensive at −1.1 V for the measurements with Pb(II) present). In the absence of Pb(II), hydrogen evolution is significantly more intensive for the bare SPE compared with SbFSPE (Figure 1). The background signal is low for both sensors in the potential range around the Pb(II) peak potential. There is a well-defined stripping signal for Pb(II) around −0.5 V for both the SbFSPE and bare SPE sensors. The advantage of using the Sb-film electrode compared to, e.g., the Bi-film electrode lies in its low oxidation stripping signal for Sb(III), as seen at a potential of approx. −0.15 V (for Bi(III) it would be significantly more intensive) [10,18,23]. Moreover, a similar Pb(II) stripping signal (peak height) was measured for both substrates indicating a similar sensitivity for the bare SPE and SbFSPE (also seen from the calibration plot slopes presented below). Similar was previously reported for Pb(II) using GCE [16], when 0.5 mg/L Sb(III) was employed to modify the surface. Interestingly, the influence of a relatively high Pb(II) concentration (in the case shown in Figure 1) on the reduced hydrogen evolution for the bare SPE is also present. In this case the intensity of hydrogen evolution is comparable to the SbFSPE, when Pb(II) was present in the solution.

The relatively low intensity peak, which is clearly expressed for bare SPE and SbFSPE at 0.15 V (as seen from the insert in Figure 1, for blank SbFSPE it develops at about 0.20 V), is most likely related to the oxidation of silver to form silver chloride (residue of silver that remained on the working electrode as reported in Ref. [34]). This peak was shown to always be present in such stripping analyses (see Figure S2 in the Supporting Information).

#### 3.1.1. LOD and LOQ Determination

The limit of detection (LOD) and limit of quantification (LOQ) were determined experimentally based on the signal-to-noise ratio (S/N) obtained by performing a SWASV measurement by successively injecting trace amounts of Pb(II) and measuring the current response in voltammograms. LOD and LOQ were determined based on the S/N ratio at a certain Pb(II) concentration, for which S/N ≥ 3 and S/N ≥ 10, respectively [35]. Signal S is the Pb(II) stripping peak height and the noise is the background noise (determined as the difference between the highest and the lowest point in the background contribution [29], evaluated in the potential region more positive and more negative around the developed Pb(II) stripping peak).

For SbFSPE, the determined LOD was 1.5 µg/L (at S/N = 4.6) and the LOQ was 3.0 µg/L (at S/N = 12.3). For bare SPE, the LOD and LOQ were determined to be 0.5 µg/L (at S/N = 3.5) and 1.5 (at S/N = 11.5), respectively. Considering the above results, it can be concluded that the formation of the in situ SbFSPE increases the LOD and LOQ values.

#### 3.1.2. Linearity

**Figure 2:**
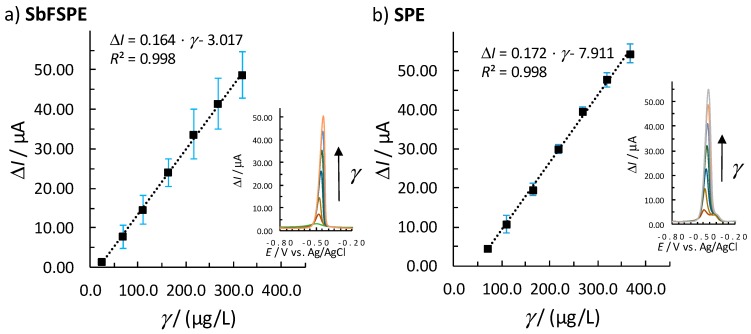
The linear Pb(II) concentration range measured in 0.01 M HCl for (**a**) SbFSPE (0.5 mg/L Sb(III)) and (**b**) bare SPE sensors; the error bars represent the standard deviation. Corresponding voltammograms with increasing Pb(II) concentrations (one out of three measurements) are shown in the insets. *E*_acc_ of –1.1 V and *t*_acc_ of 60 s were employed.

Figure 2 shows the concentration range for Pb(II) determination, regarding which the response (Δ*I*) was fitted with the linear calibration plot using the linear least squares regression method. Linearity was confirmed if a correlation coefficient of *R*^2^ > 0.99 was obtained. Moreover, to accept the linear concentration range, *y*-residuals (*y*_measured_ − *y*_model_, i.e., Δ*I*_measured_ − Δ*I*_model_) needed to be randomly distributed with increasing Pb(II) concentration. Finally, to accept linearity in a certain concentration range, the data also needed to pass the quality coefficient test with a QC < 5.0%. The normal distribution was also checked by the quantile-quantile or Q-Q plot [36].

The method showed a Pb(II) concentration linearity in the 24.0–319.1 µg/L range when using SbFSPE (Figure 2a), and in the 69.8–368.4 µg/L range when using bare SPE (Figure 2b). For bare SPE, the 5.0–69.8 µg/L concentration range was also checked to determine if a second linearity existed, but the presence of such was not determined (the same was also checked for SbFSPE for concentrations lower than 24.0 µg/L and such linearity was not determined). Measurements were performed in triplicates; each point shown is an average signal and the corresponding error bars represent the standard deviation at each concentration point measured. The determined standard deviations are not large for the measurements with bare SPE, whereas they are relatively large for a single response at a certain concentration point for SbFSPE (the standard deviations also increase with increasing Pb(II) concentration). This could indicate a heteroscedastic data distribution and the need for the weighted regression. However, in the performed analyses three new sensors were employed, which could potentially mean that the sensors exhibit minor differences (e.g., a minor composition difference, slightly different measurement areas [34], etc.). As the slope and the intercept change when successive linear calibration plots are performed with new SbFSPE sensors (also seen in Figure 4a), it is rather recommended to employ the multiple standard addition method for the determination of an unknown Pb(II) concentration instead of using the calibration plot method.. However, by using new sensors the linear range is preserved (Figure S3 in the Supporting information). Moreover, compared to the results reported in Refs. [12,13], it can be concluded that by employing shorter *t*_accu_ the linear range substantially increases, but the sensitivity decreases (evaluated from the slope of the calibration curve). 

**Figure 3:**
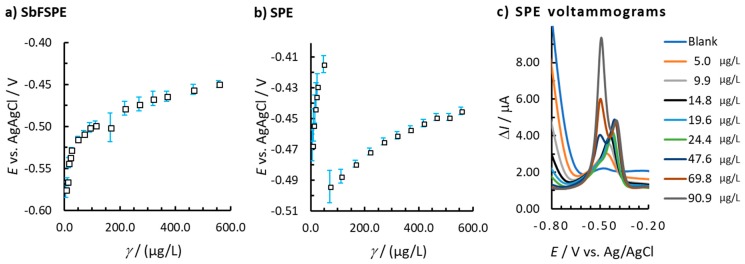
(**a**) The change in peak potential vs. Pb(II) concentration for (**a**) SbFSPE and (**b**) bare SPE. (**c**) the corresponding voltammograms for bare SPE; the error bars represent the standard deviation. The same experimental conditions were employed as in Figure 2.

The peak potential for Pb(II) is in a potential range from –0.575 V to −0.449 V for SbFSPE, and in a potential range from −0.493 to −0.414 V for bare SPE by increasing the Pb(II) concentration from 5.0 to 555.6 µg/L. In general, the peak potential shifts to more positive potentials with increasing Pb(II) concentration for both bare SPE and SbFSPE systems (Figure 3), apart from one break in the case of bare SPE when the Pb(II) concentration increases from 47.6 µg/L to 69.8 µg/L (Figure 3b). The reason for such a feature lies in the formation of two peaks (as seen in Figure 3c). At Pb(II) concentrations higher than 69.8 µg/L, the peak at more negative potentials prevails, which is then used as a representative signal and for this reason the method linearity starts at that concentration point [13].

#### 3.1.3. Accuracy and Precision

**Figure 4:**
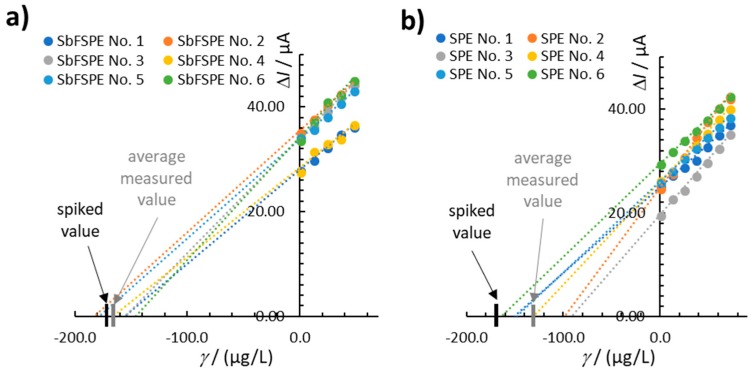
Representation of the accuracy and precision by employing the multiple standard addition method for six new electrodes used for the first time in 0.01 M HCl solution spiked with 169.1 µg/L Pb(II): (**a**) SbFSPE and (**b**) bare SPE. The same experimental conditions were employed as in Figure 2.

The accuracy was evaluated by spiking 169.1 µg/L Pb(II) in 0.01 M HCl solution using both SbFSPE and bare SPE electrodes (both sensors were shown to have a linear response at this concentration). Figure 4 shows the results obtained by employing the multiple standard addition method using 12 completely new sensor electrodes (6 for analyses with SbFSPE and 6 for bare SPE). The measured (determined) value of the Pb(II) concentration should be considered as the value where the curve crosses the x-axis in Figure 4. The analytical method using SbFSPE for Pb(II) determination seems to be more accurate (the difference between the average measured value and the true value is lower) compared with that for bare SPE (Figure 4a vs. Figure 4b) when using single sensors for the first time. The average recovery for SbFSPE was 97.0%, whereas the average recovery for bare SPE was 77.4%. The accuracy was also evaluated using a Student’s two-tailed *t*-test at 95% confidence. According to that test, the *t* values were 0.87 and 3.14 for SbFSPE and bare SPE, respectively. Therefore, according to the desired *t*^exp^ < *t*^tab^ (0.05; 5) = 2.57, SbFSPE reported accurate results, whereas bare SPE did not (as *t*^exp^ = 3.14 > *t*^tab^ = 2.57) for the first use of the respective sensor.

Higher precision was also determined in the case of SbFSPE compared with bare SPE when performing the first analysis with six new sensors. This can also be seen in Figure 4b, where the measured values were more intensively scattered around the average value compared with that in Figure 4a. The RSD values for SbFSPE and bare SPE were 8.7 % (*n* = 6) and 22.7% (*n* = 6), respectively.

Based on these results, we can conclude that the method where the in situ Sb-film is formed (SbFSPE) alongside the Pb pre-concentration is more accurate and precise when compared to the method using bare SPE. It is important to note once more that this is true for the first-time use of the sensor. After reuse, different performances of the electrodes were determined, which will be further explained below.

#### 3.1.4. Reusability of SbFSPE and Bare SPE Sensors

One of the most important (and yet to be answered) questions with regard to SPE sensor use is how many consecutive analyses can be conducted using one electrode (reusability). SPE electrodes are in general meant to be disposable, but this does not mean that they can only be used to conduct one analysis. As already mentioned in the introductory section, this is the first study to determine the number of successive (and reliable) measurements using these sensors before discarding them.

To answer this question, the same analyses as employed for evaluating accuracy and precision in Section 3.1.3 were performed in sequence by using the same (already used) sensor. This analysis (the multiple standard addition method) was repeated five times with six sensors for both SbFSPE and bare SPE (the results are presented in Figure S4 in the Supporting information).

**Figure 5:**
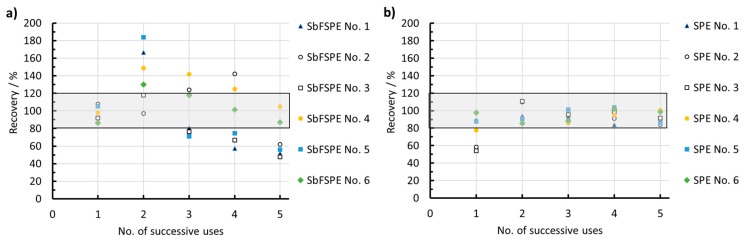
Recovery vs. the number of successive multiple standard addition analyses using the same sensor (repeated for six (**a**) SbFSPE and six (**b**) bare SPE sensors). The same experimental conditions were employed as in Figure 2.

Figure 5 shows how the performance of SbFSPE and bare SPE sensors change in terms of recovery values when using the same sensor for five successive determinations of Pb(II) by the multiple standard addition method. The 0.01 M HCl solution was spiked with a 169.1 µg/L Pb(II) concentration. As reported above, SbFSPE produced accurate and precise results when a completely new sensor was employed (i.e., it was used for the first time). When the same SbFSPE sensors were used for the second time, four of six SbFSPE sensors showed results that were outside the 80–120% recovery values, and thus produced inaccurate results (Figure 5a). With further use (from the third to fifth instance of use), the SbFSPE sensors mainly produced inaccurate results outside the 80–120% recovery range (the method was deemed to be accurate when the recovery was between 80–120% [37]).

In contrast to the SbFSPE sensor, the first use of the bare SPE sensor in general produced inaccurate results (three out of six sensors reported recovery values outside the 80–120% recovery range and the determined average values of the six sensors used did not pass the *t*-test, as reported above), further use of the same sensors resulted in recovery ranges inside the 80–120% recovery range limits. It seems that the bare SPE sensor in this medium needs some pre-activation to provide accurate results. The change in electrochemical performance with successive use is also demonstrated (and further discussed) below in Section 3.2 using the EIS technique.

### 3.2. Electrochemical Impedance Spectroscopy Measurements

**Figure 6:**
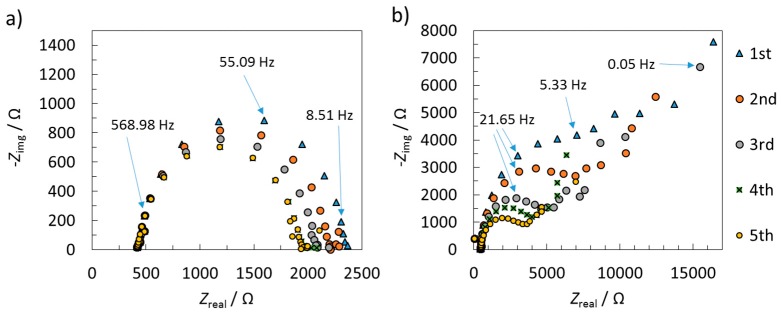
Successive EIS measurements for (**a**) SbFSPE and (**b**) bare SPE. EIS measurements were performed at −1.1 V after a pre-concentration step (*E*_acc_ = −1.1 V, *t*_acc_ = 60 s) in 0.01 M HCl containing 169.1 µg/L Pb(II). In the case of SbFSPE a 0.5 mg/L Sb(III) was employed.

Figure 6 shows the Nyquist spectra measured after the accumulation step (repeated five times; at *E*_accu_ = −1.1 V, *t*_accu_ = 60 s) and cleaning step. Using such an approach, electrochemical evaluation of the change in sensor performance after reuse can be reported. The shape (a semicircle) of the spectra for SbFSPE indicates a charge-transfer-controlled reaction (a kinetic-controlled reaction). On the other hand, the Nyquist spectra for the bare SPE sensor show that the system is also under diffusion control (besides kinetic control), as the measured curve deviates from a semicircle in the low frequency region (at high *Z*_real_) in a manner typical of a diffusion-controlled reaction [38,39].

Figure 6a shows that the electrochemical performance of the SbFSPE sensor changes with successive use, with a decrease in the resistance of the system after each use (*Z*_real_ decreases with successive use). However, this change with successive use is even more significant for bare SPE (Figure 6b vs. Figure 6a). The latter could explain the fact that the bare SPE sensor became more useful after its first use (the recovery values became acceptable, Figure 5). Moreover, the decrease in the interfacial electron transfer resistance has previously been explained as an increase in the sensitivity of the method [39,40].

### 3.3. Surface Morphology And Topography Characterisation

**Figure 7:**
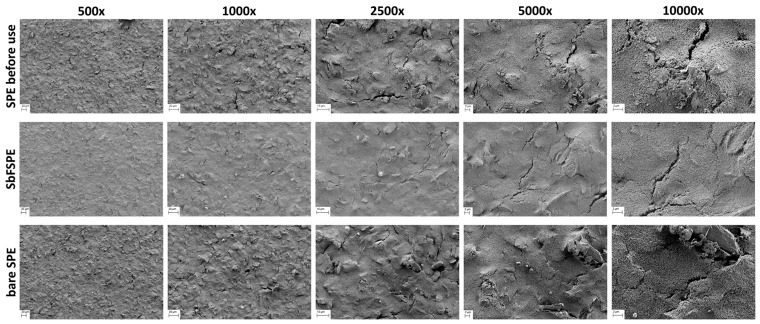
SEM images measured on the surface of three different sensors: top row: SPE sensors before use, middle row: SbFSPE sensors after being used five times, and bottom row: bare SPE after being used five times by employing the multiple standard addition method. In the case of used samples, the same experimental conditions were employed as in Figure 2.

Figure 7 shows representative surface morphologies of the working electrode before and after five-time use (bare SPE and SbFSPE sensors). The micrographs shown are only for those electrodes that produced results, which are reported in Figure 4 (measurements for three out of six sensors are presented). The surface morphology of all samples was evaluated at various magnifications to observe potential features and their distribution on representative scales. The surface morphologies of the bare SPE sensor before use and the SbFSPE sensor after five-time use, are similar regardless of the respective micrographs’ magnification. Both exhibit some minor cracks in the surface, which seem to be evenly distributed over the surface. On the other hand, the bare SPE sensor after five-time use shows a different morphology, which is the most evident at higher magnifications (2500× and higher). This electrode exhibits a higher surface roughness compared to the other two samples and partially exposed bigger materials pieces, which are not visible on neither the SPE sensor before use not the SbFSPE sensor after five-time use. Furthermore, it seems that at certain places on the working electrode surface, the carbon-based working material starts to peel off. This effect is more expressed for the bare SPE sensor. Based on these findings, we conducted AFM measurements to get further insight into potential differences on an even smaller scale (compared to the magnification of 10,000×).

**Figure 8:**
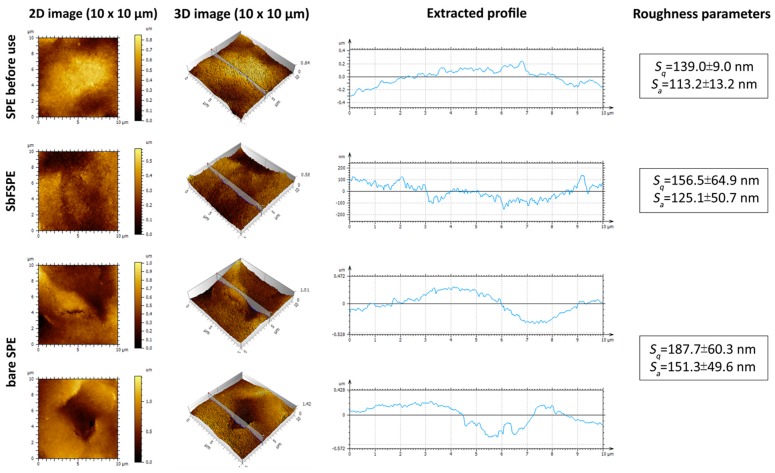
AFM images, extracted profiles, and roughness parameters of (**top row**) the SPE sensor before use, (**second row**) the SbFSPE sensor after five successive uses, and (**bottom two rows**) a bare SPE after five successive uses, employing the multiple standard addition method. In the case of used samples, the same experimental conditions were employed as in Figure 2.

Figure 8 shows the sample topographies in 2D and 3D, and the corresponding extracted profiles for the SPE sensor before use, as well as the bare SPE sensors and the SbFSPE sensors after five successive uses. The analyses were performed on the same samples as used for the SEM analyses. For Figure 8, the most representative AFM images were chosen (measured with dimensions of 10 μm × 10 μm), which were further used to calculate (on the basis of 12 measurements at different places and on 3 sensors) the most commonly evaluated roughness parameters based on the ISO 25178 standard, (i.e., arithmetical mean height of the surface—*S*_a_, and the root mean square height of the surface—*S*_q_). *S*_a_ is based on general surface roughness; the higher the *S*_a_ value, the rougher the surface is [41], whereas *S*_q_ presents the most commonly used height parameter, which involves only the statistical distribution of height values along the *z* axis.

Based on the obtained topography images, and even more on the calculated roughness parameters (last column in Figure 8), the SPE sensor before use and the SbFSPE sensor after five-time use, have a different topography compared with the bare SPE sensor after five-time use. For example, the obtained images reveal a small crack in the case of the bare SPE sensor after five-time use. Similar features were found at various parts of the same sample (only one is shown herein). This result is also in agreement with the above discussed results from the SEM measurements, where similar (more pronounced in the case of bare SPE sensor after five-time use compared to the SbFSPE sensor after five-time use and the SPE sensor before use) cracks were observed over the whole surface. Furthermore, the AFM images show a higher frequency of “valleys and mountains” for the bare SPE sensor after five-time use, which contributes to the higher roughness parameters calculated for this sample. Furthermore, Figure 8 also shows another typical defect formed on the working electrode surface of bare SPE sensor after five successive uses—a hole approx. 0.6 µm deep. 

In conclusion, both methods used for evaluation of the sample topographies before and after five-time use (SEM and AFM), confirm that the reusability of bare SPE induces more significant changes in the working electrode surface compared to SbFSPE after the same number of uses.

### 3.4. Interference Study

The interference effect of different compounds (probable interferents) on the Pb(II) stripping signal, which could possibly be present in real water samples, was checked by preparing solutions with a Pb(II):interferent mass concentration ratios of 1:1, 1:10, and 1:100 [15]. The analysis was performed for SbFSPE as it showed accurate and precise results when used for the first time in 0.01 M HCl solution (see Section 3.1.3). The calculated influence as to how much the interferents increase or decrease the Pb(II) stripping signal is summarised in Table 2. By means of this approach it was possible to clearly show which interferent has an influence on the Pb(II) stripping signal (if any influence is present, the ratio deviates from 1). The influence can be observed by either an increase in the Pb(II) peak (a positive effect) or a decrease in the Pb(II) peak (a negative effect).

In the case of equal amounts of Cd(II) and Pb(II), i.e., a 1:1 ratio, a more intensive Pb(II) signal was obtained. On the other hand, at higher Cd(II) concentrations the Pb(II) signal decreased. At a 1:100 Pb(II):Cd(II) ratio, the Pb(II) and Cd(II) stripping peaks overlapped, and therefore Pb(II) cannot be detected (Figure S5 in the Supporting information). 

Zn(II), Fe(II), Na(I), As(III), K(I), Ca(II), and Mg(II), and NO_3_^−^ have a minor influence on the Pb(II). As expected, Bi(III), Cu(II), Sn(II), and Hg(II) significantly influence the Pb(II) stripping signal when they are employed at high concentrations, as these ions are used to form the in situ electrode.

### 3.5. Analytical Application of Real Water Samples

The applicability of the SbFSPE sensor was also checked with regard to a real water sample (as it showed accurate and precise results when used for the first time in the model 0.01 M HCl solution (see Section 3.1.4)). As shown above, the standard addition method had to be employed when using the SbFSPE sensor. Moreover, by using this procedure the matrix effects in the analysis of the real water sample are minimised. The applicability of the SbFSPE sensor was tested regarding a real river water sample in order to check for possible interference effects. Five replicate measurements using the multiple standard addition method were performed, each time using a new sensor and freshly prepared solutions.

**Figure 9:**
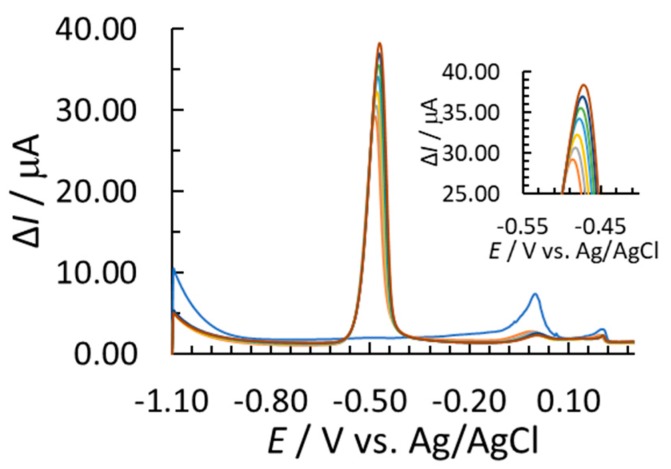
Voltammograms measured using SbFSPE (0.5 mg/L (Sb(III)) for the real river water sample without and with the successive addition of Pb(II) (*E*_acc_ = −1.1 V, *t*_acc_ = 60 s).

No stripping Pb(II) signal was detected for the river water sample (the results are not shown herein), therefore the Pb(II) concentration in this sample was below the LOD of the method. The same was confirmed by the ICP-OES referential technique. Next, this water sample was spiked with 169.1 µg/L Pb(II), and the multiple standard addition method was employed to determine the Pb(II) concentration to check for any possible interference effect. A well-defined Pb(II) stripping peaks were developed (Figure 9). The interference effect was assessed based on a two-sample *t*-test (two-tailed and small sample populations) for equal means. The river water sample and the model solution (prepared with ultra-pure water, as was done for the method validation study presented above) had statistically equal variances (assessed on the basis of a two-tailed *F*-test at 95% confidence). Moreover, based on the calculated experimental *t*-value for the two sample means with statistically equal variances at 95% confidence, which was lower than the critical *t*-value (as *t*^exp^ = 0.68 < *t*^tab^ = 2.30), it can be concluded that the SbFSPE sensor does not suffer from an interference effect from the river water sample. Moreover, replicate measurements reported precision in terms of RSD as 8.1% (*n* = 5). Furthermore, no significant statistical difference was identified when comparing the results of this spiked river water sample with the referential ICP-OES method (using the *t*-test) at 95% confidence, thus providing statistically equal results. These results demonstrate the suitability of the SbFSPE sensor for real water sample analysis.

## 4. Conclusions

Screen-printed electrode (SPE) sensors are, in general, meant to be disposable. However, possible reuse—for at least a few times before being discarded—is frequently not, if at all, reported. In this work, SPE with a carbon-based working electrode was employed for Pb(II) trace analysis. The method’s analytical performance in 0.01 M HCl solution with an accumulation potential of −1.1 V vs. Ag/AgCl and an accumulation time of 60 s was validated. The carbon-based surface of the working electrode was employed in either an unmodified (bare SPE sensor) or Sb-film modified (formed in situ, SbFSPE sensor) form. It was found that the detection and quantification limits were lower when employing the bare SPE sensor. Linear concentration ranges were 69.8–368.4 µg/L and 24.0–319.1 µg/L, for the bare SPE and SbFSPE sensors, respectively. The accuracy and precision were significantly better for SbFSPE compared with SPE when these sensors were employed for the first time. However, with successive use of the same sensors, the accuracy and precision became worse for SbFSPE, whereas bare SPE produced even more accurate results when used for the second time (even up to the fifth use). This implies that bare SPE in such medium needs pre-activation, which was ascertained by the first analysis. Electrochemical impedance spectroscopy measurements showed that successive use of SbFSPE and bare SPE sensors change their electrochemical properties. It was also shown that the system was under kinetic-control in the case of SbFSPE, whereas bare SPE followed a kinetic- and diffusion-controlled process. AFM and SEM showed that successive use degrades the surface of both sensors (SbFSPE and bare SPE) after multiple use. In the interference study for SbFSPE, the presence of Cd(II) increased the Pb(II) signal when present at equal concentrations, whereas at a 10-time higher Cd(II) concentration compared to Pb(II), the Pb(II) signal decreased. When Zn(II), As(III), and Fe(II), Na(I), K(I), Ca(II), and Mg(II), and NO_3_^−^ ions were present individually, along with Pb(II), no significant change in the Pb(II) signal was found. On the other hand, Bi(III), Cu(II), Sn(II), and Hg(II) significantly influenced the Pb(II) stripping signal. The applicability of the SbFSPE sensor was also proven for the real sample analysis, i.e. river water, which provided accurate and precise results. This work, therefore, reports an analytical method that is inexpensive, produces a relatively fast analysis, and enables use on-site.

## Figures and Tables

**Table 1:** Comparison of different SPEs performances for Pb(II) determination.

Working Electrode	*E*acc [V]	Method	Linear Concentration Range (µg/L)	LOD (µg/L)	Reference
SbSPCE	−1.2 −0.7	DPASV	16.8–62.6 16.1–55.5	5.0 4.8	[12]
SbSPCE SbSPCE-CNF SbSPCE-MWCNT SbSPCE-GPH	−1.5	DPASV	17.5–100.9 6.9–100.9 14.7–100.9 28.8–100.9	5.3 2.1 4.4 8.6	[13]
Bi-SbSPE	−1.3	SWASV	0.1–90	0.07	[24]
SbSPE Sb_2_O_3_-SPE Sb^III^(C_2_O_4_)OH-SPE SnO_2_/Sb_2_O_5_-SPE	−1.4 (vs. SCE) −1.4 (vs. SCE) −1.4 (vs. SCE) −1.5 (vs. SCE)	DPASV	5–45	1.0 0.9 1.1 1.1	[22]
SbF-GO-SPCE	−1.4	SWASV	0.1–1.3 (µM)	0.026 (µM)	[27]
*Ex situ*-SbSPCE-CNF	−1.4	DPASV	13.2–200.8	4.0	[28]
SbFSPE Bare SPE	−1.1	SWASV	24.0–319.1 69.8–368.4	1.50 0.50	this work

SbSPCE: antimony screen-printed carbon electrode, SbSPCE-CNF: antimony film electrode prepared from carbon nanofibers modified screen-printed electrodes, SbSPCE-MWCNT: antimony film electrode prepared from multi-walled carbon nanotubes modified screen-printed electrode, SbSPCE-GPH: antimony film electrode prepared from graphene modified screen-printed electrode, Bi-SbSPE: bismuth and antimony screen-printed electrode, SbSPE: antimony screen-printed electrode, Sb_2_O_3_-SPE: screen-printed electrode bulk-modified with Sb_2_O_3_, Sb^III^(C_2_O_4_)OH-SPE: screen-printed electrode bulk-modified with Sb^III^(C_2_O_4_)OH, SnO_2_/Sb_2_O_5_-SPE: screen-printed electrode bulk-modified with SnO_2_/Sb_2_O_5_, SbF-GO-SPCE: antimony-graphene oxide modified screen-printed carbon electrode, DPASV: differential pulse anodic stripping voltammetry.

**Table 2:** The influence of different compounds on the Pb(II) peak height: the values are calculated as the $\mathrm{change}\;\mathrm{in}\;\%=\left(\frac{I_{\mathrm{interferent}}-I_{\mathrm{Pb}\left(\mathrm{II}\right)}}{I_{\mathrm{Pb}\left(\mathrm{II}\right)}}\right)\cdot 100\%$, where *I*_interferent_ and *I*_Pb(II)_ are the stripping signals for Pb(II) at 200.0 µg/L, with and without the presence of interferent, respectively, in 0.01 M HCl solution [15]. Measurements were performed using the same experimental conditions as for Figure 2.

Interferent	Mass Concentration Ratio Pb(II):Interferent		
	1:1	1:10	1:100
Cd(II)	13.4	−31.1	ND
Zn(II)	3.5	9.1	10.6
Fe(II)	0.8	1.5	−5.6
As(III)	0.6	1.1	6.0
Na(I)	−0.1	−1.0	−2.8
K(I)	−0.1	−0.5	−2.9
Ca(II)	−0.6	−0.9	−2.9
Mg(II)	−1.2	−1.4	−3.4
Bi(III)	43.2	28.9	19.5
Cu(II)	−78.8	−45.2	43.3
Sn(II)	1.8	−0.7	77.5
Hg(II)	12.6	59.7	115.8
NO_3_^–^	0.4	−0.4	−3.0

ND—the Pb(II) peak was not detected.

## Supplementary Materials

The following are available online at http://www.mdpi.com/1424-8220/18/11/3976/s1. Figure S1: SPE electrode immersed in 1.0 M KCl containing 10 mM K_3_[FeCN_6_]: (a) CV at different scan rates, (b) cathodic current peak vs. scan rate, and (c) anodic current peak vs. scan rate. Figure S2: The development of the low intensity peak using 6 new sensors for (a) blank SbFSPE sensor (0.5 mg/L Sb(III)) and (b) blank SPE sensor measured in 0.01 M HCl (*E*_acc_ = −1.1 V and *t*_acc_ = 60 s). Figure S3: Individual calibration plots for SbFSPE. The rectangles represent the linear range, and the circles represent measurements outside the linear concentration range. Measurements were performed in 0.01 M HCl using *E*_acc_ = −1.1 V and *t*_acc_ = 60 s, each time a new sensor was employed. Figure S4: Five consecutive multiple standard addition method analyses using (a) six new SbFSPE sensors and (b) six new bare SPE sensors, for Pb(II) determination. The 0.01 M HCl was spiked with 169.1 µg/L Pb(II). Figure S5: SWASV voltammograms measured using SbFSPE in 0.01 M HCl containing 200.0 µg/L Pb(II) with and without possible interferents at a mass concentration ratio of 1:1, 1:10, and 1:100 relative to Pb(II).

## References

[1] Lu Y., Liang X., Niyungeko C., Zhou J., Xu J., Tian G. (2018). A review of the identification and detection of heavy metal ions in the environment by voltammetry. Talanta.

[2] Jovanovski V., Hrastnik N.I., Hočevar S.B. (2015). Copper film electrode for anodic stripping voltammetric determination of trace mercury and lead. Electrochem. Commun..

[3] Minamata Convention on Mercury. http://www.mercuryconvention.org/.

[4] Wang J., Lu J., Hocevar S.B., Farias P.A.M., Ogorevc B. (2000). Bismuth-Coated Carbon Electrodes for Anodic Stripping Voltammetry. Anal. Chem..

[5] Jovanovski V., Hočevar S.B., Ogorevc B. (2017). Bismuth electrodes in contemporary electroanalysis. Curr. Opin. Electrochem..

[6] Arduini F., Calvo J.Q., Palleschi G., Moscone D., Amine A. (2010). Bismuth-modified electrodes for lead detection. TrAC Trends Anal. Chem..

[7] Koshy O., Pottathara Y.B., Thomas S., Petovar B., Finšgar M. (2017). A flexible, disposable hydrogen peroxide sensor on graphene nanoplatelet-coated cellulose. Curr. Anal. Chem..

[8] Serrano N., Díaz-Cruz J.M., Ariño C., Esteban M. (2016). Antimony-based electrodes for analytical determinations. TrAC Trends Anal. Chem..

[9] Pauliukaite R., Metelka R., Švancara I., Królicka A., Bobrowski A., Norkus E., Kalcher K., Vytřas K. (2004). Screen-printed carbon electrodes bulk-modified with Bi_2_O_3_ OR Sb_2_O_3_ for trace determination of some heavy metals. Sci. Pap. Univ. Pardubice Ser. A.

[10] Hocevar S.B., Švancara I., Ogorevc B., Vytřas K. (2007). Antimony Film Electrode for Electrochemical Stripping Analysis. Anal. Chem..

[11] Pérez-Ràfols C., Trechera P., Serrano N., Díaz-Cruz J.M., Ariño C., Esteban M. (2017). Determination of Pd(II) using an antimony film coated on a screen-printed electrode by adsorptive stripping voltammetry. Talanta.

[12] Sosa V., Barceló C., Serrano N., Ariño C., Díaz-Cruz J.M., Esteban M. (2015). Antimony film screen-printed carbon electrode for stripping analysis of Cd(II), Pb(II), and Cu(II) in natural samples. Anal. Chim. Acta.

[13] Pérez-Ràfols C., Serrano N., Díaz-Cruz J.M., Ariño C., Esteban M. (2016). New approaches to antimony film screen-printed electrodes using carbon-based nanomaterials substrates. Anal. Chim. Acta.

[14] Vasko J., Samo B.H., Božidar O. (2009). Ex Situ Prepared Antimony Film Electrode for Electrochemical Stripping Measurement of Heavy Metal Ions. Electroanalysis.

[15] Slavec M., Hocevar S.B., Baldrianova L., Tesarova E., Svancara I., Ogorevc B., Vytras K. (2010). Antimony Film Microelectrode for Anodic Stripping Measurement of Cadmium(II), Lead(II) and Copper(II). Electroanalysis.

[16] Sebez B., Ogorevc B., Hocevar S.B., Veber M. (2013). Functioning of antimony film electrode in acid media under cyclic and anodic stripping voltammetry conditions. Anal. Chim. Acta.

[17] Guzsvány V., Nakajima H., Soh N., Nakano K., Imato T. (2010). Antimony-film electrode for the determination of trace metals by sequential-injection analysis/anodic stripping voltammetry. Anal. Chim. Acta.

[18] Tesarova E., Baldrianova L., Hocevar S.B., Svancara I., Vytras K., Ogorevc B. (2009). Anodic stripping voltammetric measurement of trace heavy metals at antimony film carbon paste electrode. Electrochim. Acta.

[19] Ashrafi A.M., Vytřas K. (2012). New procedures for voltammetric determination of copper (II) using antimony film-coated carbon paste electrodes. Electrochim. Acta.

[20] Li M., Li D.-W., Xiu G., Long Y.-T. (2017). Applications of screen-printed electrodes in current environmental analysis. Curr. Opin. Electrochem..

[21] Rawlinson S., McLister A., Kanyong P., Davis J. (2018). Rapid determination of salicylic acid at screen printed electrodes. Microchem. J..

[22] Maczuga M., Economou A., Bobrowski A., Prodromidis M.I. (2013). Novel screen-printed antimony and tin voltammetric sensors for anodic stripping detection of Pb(II) and Cd(II). Electrochim. Acta.

[23] Niu X., Zhao H., Lan M. (2011). Disposable screen-printed antimony film electrode modified with carbon nanotubes/ionic liquid for electrochemical stripping measurement. Electrochim. Acta.

[24] Chen C., Niu X., Chai Y., Zhao H., Lan M., Zhu Y., Wei G. (2013). Determination of Lead(II) Using Screen-Printed Bismuth-Antimony Film Electrode. Electroanalysis.

[25] Puy-Llovera J., Pérez-Ràfols C., Serrano N., Díaz-Cruz J.M., Ariño C., Esteban M. (2017). Selenocystine modified screen-printed electrode as an alternative sensor for the voltammetric determination of metal ions. Talanta.

[26] Yáñez-Sedeño P., Campuzano S., Pingarrón J.M. (2018). Integrated Affinity Biosensing Platforms on Screen-Printed Electrodes Electrografted with Diazonium Salts. Sensors.

[27] Ruengpirasiri P., Punrat E., Chailapakul O., Chuanuwatanakul S. (2017). Graphene Oxide-Modified Electrode Coated with in-situ Antimony Film for the Simultaneous Determination of Heavy Metals by Sequential Injection-Anodic Stripping Voltammetry. Electroanalysis.

[28] Pérez-Ràfols C., Serrano N., Díaz-Cruz J.M., Ariño C., Esteban M. (2017). A screen-printed voltammetric electronic tongue for the analysis of complex mixtures of metal ions. Sens. Actuators B Chem..

[29] Massart D.L., Vandeginste B.G.M., Buydens L.M.C., Jong S.D., Lewi P.J., Smeyers-Verbeke J. (1997). Handbook of Chemometrics and Qualimetrics: Part A.

[30] Maver U., Xhanari K., Zizek M., Korte D., Gradisnik L., Franko M., Finsgar M. (2018). A combination of interdisciplinary analytical tools for evaluation of multi-layered coatings on medical grade stainless steel for biomedical applications. Eur. J. Pharm. Biopharm..

[31] Mohan T., Findenig G., Hollbacher S., Cerny C., Ristic T., Kargl R., Spirk S., Maver U., Stana-Kleinschek K., Ribitsch V. (2014). Interaction and enrichment of protein on cationic polysaccharide surfaces. Colloids Surf. B Biointerfaces.

[32] Horcas I., Fernandez R., Gomez-Rodriguez J.M., Colchero J., Gomez-Herrero J., Baro A.M. (2007). WSXM: A software for scanning probe microscopy and a tool for nanotechnology. Rev. Sci. Instrum..

[33] Finšgar M., Petovar B. (2018). Novel in situ Bi−Sb-Film Electrodes for Trace Heavy Metal Analysis. Electroanalysis.

[34] Lee P., Lowinsohn D., Compton R. (2014). The Use of Screen-Printed Electrodes in a Proof of Concept Electrochemical Estimation of Homocysteine and Glutathione in the Presence of Cysteine Using Catechol. Sensors.

[35] ICH Validation of analytical procedures: Text and methodology Q2(R1), Harmonised Tripartite Guideline. Proceedings of the International Conference on Harmonisation of Technical Requirements for Registration of Pharmaceuticals for Human Use.

[36] Van Loco J., Elskens M., Croux C., Beernaert H. (2002). Linearity of calibration curves: Use and misuse of the correlation coefficient. Accredit.Qual. Assur..

[37] Laboratory and Scientific Section United Nations Office on Drugs and Crime (2009). Guidance for the Validation of Analytical Methodology and Calibration of Equipment Used for Testing of Illicit Drugs in Seized Materials and Biological Specimens, A Commitment to Quality and Continuous Improvement.

[38] Finšgar M., Kek Merl D. (2013). 2-Mercaptobenzoxazole as a copper corrosion inhibitor in chloride solution: Electrochemistry, 3D-profilometry, and XPS surface analysis. Corros. Sci..

[39] Petovar B., Xhanari K., Finšgar M. (2018). A detailed electrochemical impedance spectroscopy study of a bismuth-film glassy carbon electrode for trace metal analysis. Anal. Chim. Acta.

[40] Shi L., Li Y., Rong X., Wang Y., Ding S. (2017). Facile fabrication of a novel 3D graphene framework/Bi nanoparticle film for ultrasensitive electrochemical assays of heavy metal ions. Anal. Chim. Acta.

[41] Finšgar M. (2015). The first X-ray photoelectron spectroscopy surface analysis of 4-methyl-2-phenyl-imidazole adsorbed on copper. Anal. Methods.

