# Supplementary material for: Reusability of SPE and Sb-modified SPE Sensors for Trace Pb(II) Determination
Source: Sensors (Basel). 2018 Nov 15;18(11):3976. doi: 10.3390/s18113976 (PMC6263962; doi:10.3390/s18113976)
Supplement: Supplementary file 1 [file sensors-18-03976-s001.pdf]

Supporting Information

The Reversibility of the Redox System for Bare SPE

In order to check the reversibility of the SPE sensor, a cyclic voltammetry (CV) test was performed for the potassium hexacyanoferrate system (i.e. a one-electron transfer reaction). This experiment was carried out in 1.0 M KCl containing 10 mM $K_3[FeCN_6]$. The CV experiment started at 0.8 V with a negative-going potential scan until −0.3 V was reached (switching potential). Then the potential scan was reversed towards more positive potentials until the starting potential was reached. Different sweep rates (ν) were employed in sequence, i.e., 10, 20, 50, 75, 125, 150, 175, and 200 mV/s. During these measurements the solution was not stirred.

The criteria for the reversibility of this system are the following: (1) the anodic peak height (I_{pa}) and cathodic peak height (I_{pc}) increase linearly with an increase in $\sqrt{\nu}$; (2) the anodic peak potential (E_a) and cathodic peak potential (E_c) do not change with $\sqrt{\nu}$; (3) $\Delta E = E_a - E_c = 59$ mV; and (4) the I_{pa}/I_{pc} at a specific ν is 1 [28].

Figure S1a shows the change in the shape of the CV voltamogramms with an increase in ν. I_{pa} and I_{pc} increased linearly with increasing ν (Figure S1b, c) and therefore satisfy criterion 1 (as given above). On the other hand, E_a slightly shifts to more positive potentials and E_c slightly shifts to more negative potentials with increasing ν. Moreover, the ΔE values were in the range of 90–95 mV for all three measurement replications at ν = 10 mV/s, and this potential difference increased with increasing ν. Finally, the values of the ratio I_{pa}/I_{pc} were 0.89–0.95 when employing ν of 10–200 mV/s. Therefore, we can conclude that bare SPE deviates slightly from the ideal criteria 2–4 (as given above).

This testing reversibility reaction protocol cannot be repeated with SbFSPE as the Sb-film would dissolve in the anodic cycle at about −0.15 V.

Supporting Information Figures

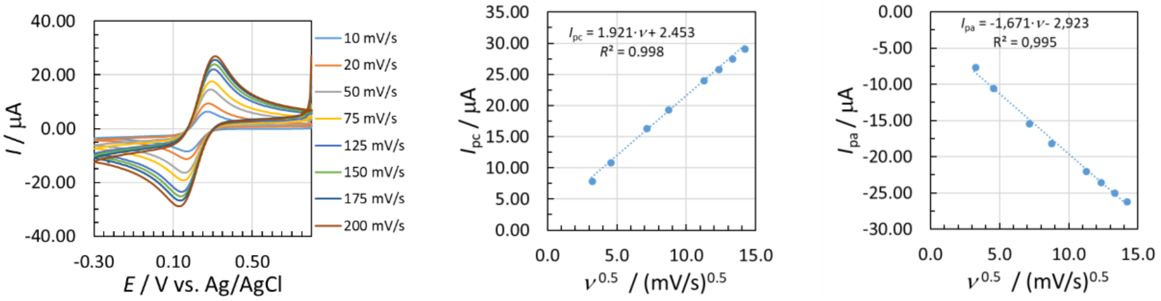

(**a**) (**b**) (**c**)

Figure S1. SPE electrode immersed in 1.0 M KCl containing 10 mM $K_3[FeCN_6]$: (**a**) CV at different scan rates, (**b**) cathodic current peak vs. scan rate, and (**c**) anodic current peak vs. scan rate.

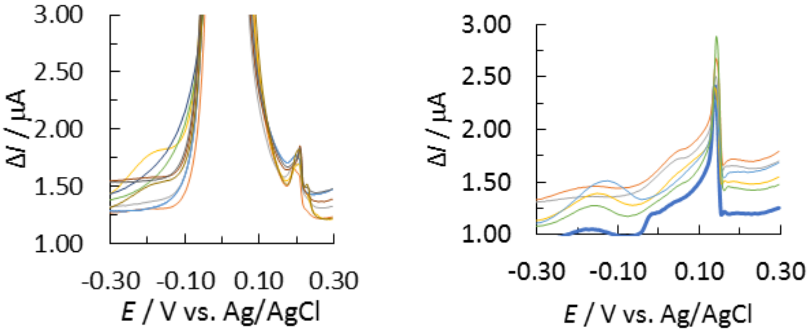

(**a**) (**b**)

Figure S2. The development of the low intensity peak using 6 new sensors for (**a**) blank SbFSPE sensor (0.5 mg/L Sb(III)) and (b) blank SPE sensor measured in 0.01 M HCl (E_{acc} = −1.1 V and t_{acc} = 60 s).

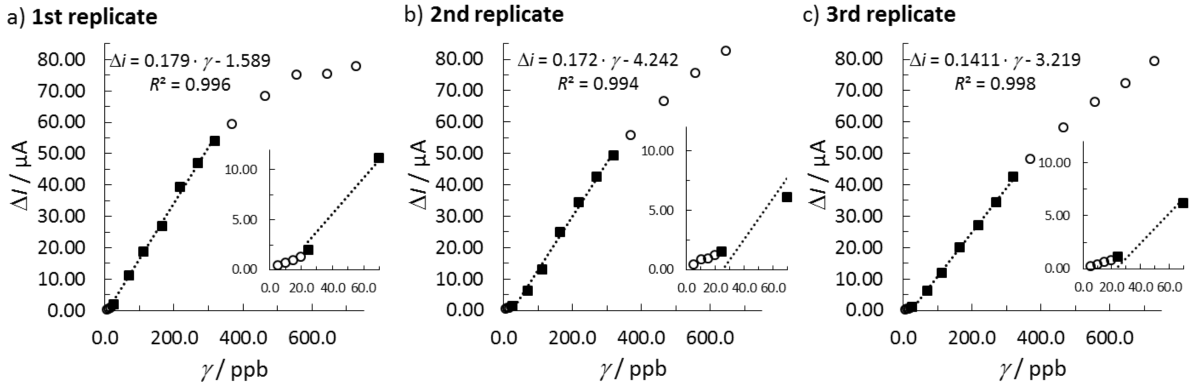

Figure S3. Individual calibration plots for SbFSPE. The rectangles represent the linear range, and the circles represent measurements outside the linear concentration range. Measurements were performed in 0.01 M HCl using E_{acc} = −1.1 V and t_{acc} = 60 s, each time a new sensor was employed.

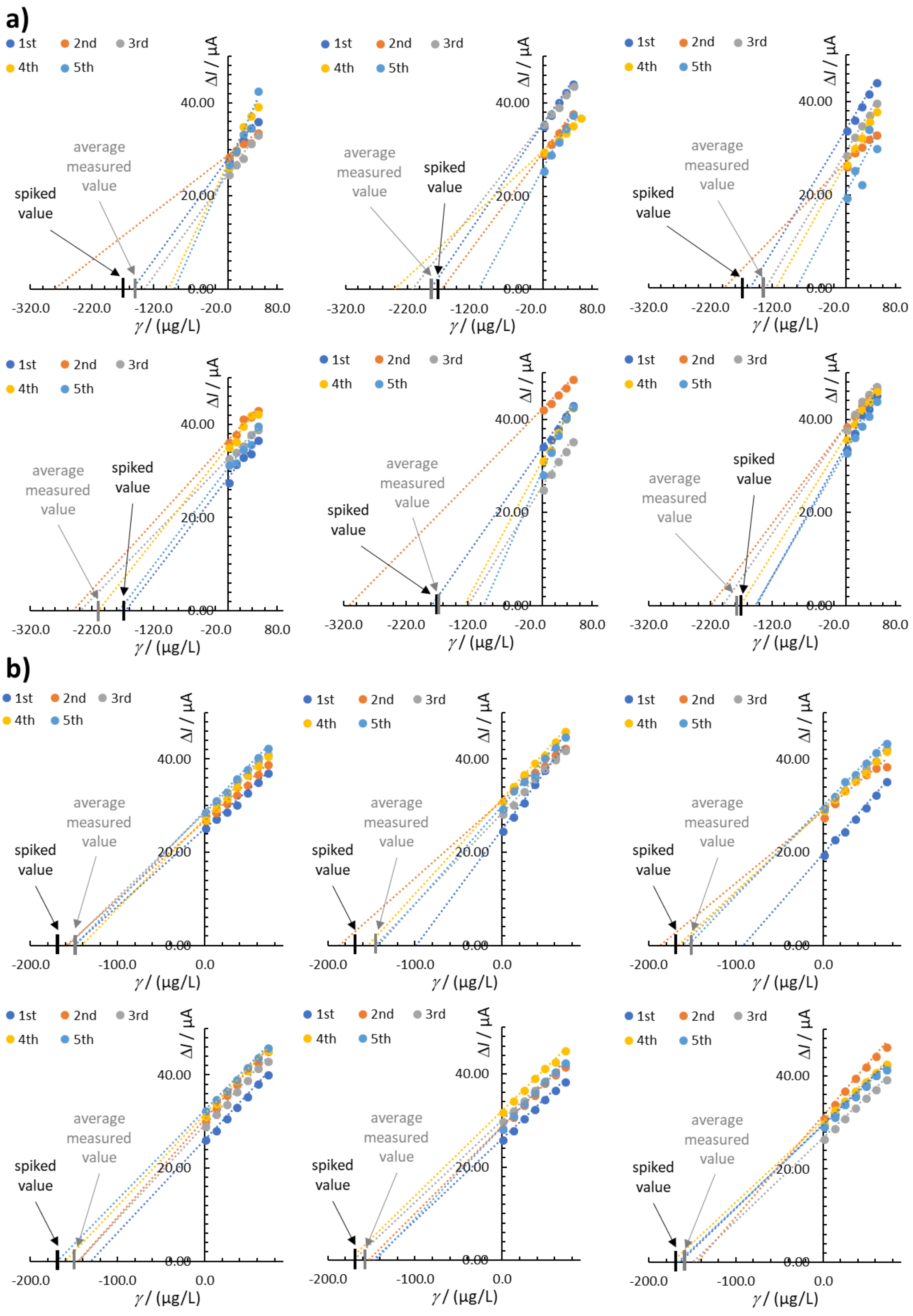

Figure S4. Five consecutive multiple standard addition method analyses using (**a**) six new SbFSPE sensors and (**b**) six new bare SPE sensors, for Pb(II) determination. The 0.01 M HCl was spiked with 169.1 µg/L Pb(II).

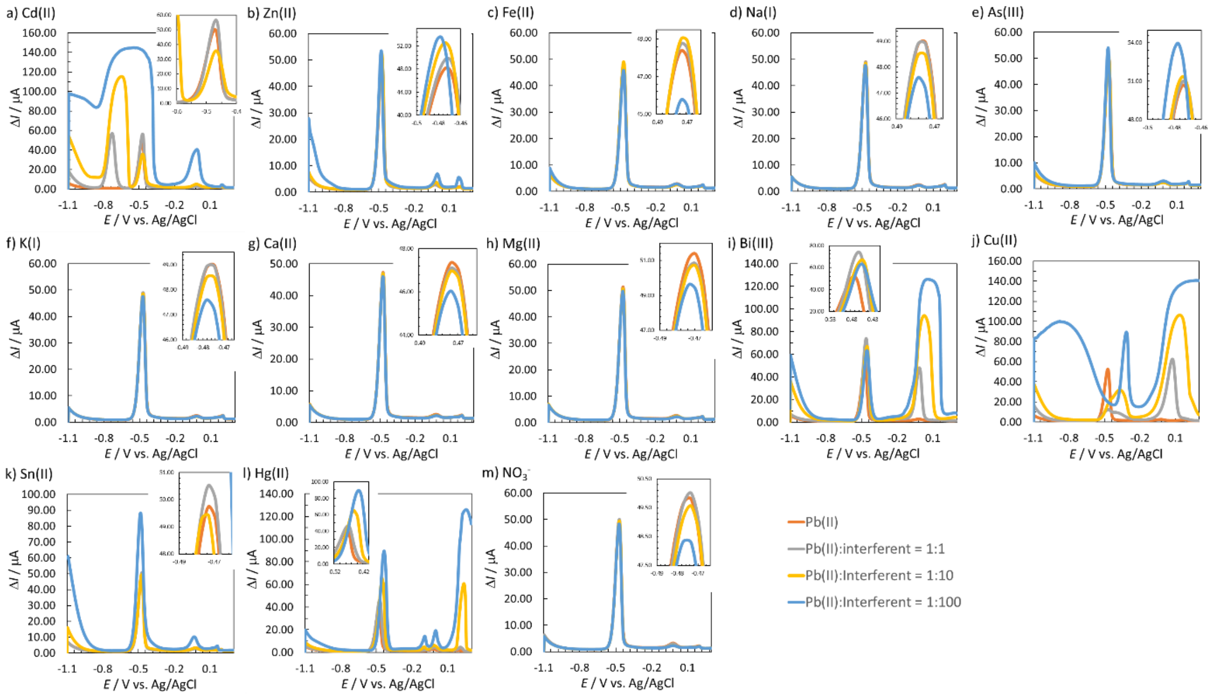

Figure S5. SWASV voltammograms measured using SbFSPE in 0.01 M HCl containing 200.0 µg/L Pb(II) with and without possible interferents at a mass concentration ratio of 1:1, 1:10, and 1:100 relative to Pb(II).
